# Assessment of methane emission traits in ewes using a laser methane detector: genetic parameters and impact on lamb weaning performance

**DOI:** 10.5194/aab-63-113-2020

**Published:** 2020-04-16

**Authors:** Jessica Reintke, Kerstin Brügemann, Tong Yin, Petra Engel, Henrik Wagner, Axel Wehrend, Sven König

**Affiliations:** 1 Institute of Animal Breeding and Pet Genetics, University of Giessen, 35390 Giessen, Germany; 2 Clinic for Obstetrics, Gynaecology and Andrology of Large and Small Animals with Veterinary Ambulance, University of Giessen, 35392 Giessen, Germany

## Abstract

The aim of the present study was to derive individual methane (${\mathrm{CH}}_{4}$) emissions in ewes separated in ${\mathrm{CH}}_{4}$ respiration and eructation traits. The generated longitudinal ${\mathrm{CH}}_{4}$ data structure was used to estimate phenotypic and genetic relationships between ewe ${\mathrm{CH}}_{4}$ records and energy efficiency indicator traits from same ewes as well as from their lambs (intergenerational perspective). In this regard, we recorded ${\mathrm{CH}}_{4}$ emissions via mobile laser methane detector (LMD) technique, body weight (EBW), backfat thickness (BFT) and body condition score (BCS) from 330 ewes (253 Merinoland (ML), 77 Rhön sheep (RH)) and their 629 lambs (478 ML, 151 RH). The interval between repeated measurements (for ewe traits and lamb body weight (LBW)) was 3 weeks during lactation. For methane concentration (µL L${}^{-1}$) determinations in the exhaled air, we considered short time measurements (3 min). Afterwards, ${\mathrm{CH}}_{4}$ emissions were portioned into a respiration and eructation fraction, based on a double normal distribution. Data preparation enabled the following ${\mathrm{CH}}_{4}$ trait definitions: mean ${\mathrm{CH}}_{4}$ concentration during respiration and eructation (${\mathrm{CH}}_{4_{r+e}}$), mean ${\mathrm{CH}}_{4}$ concentration during respiration (${\mathrm{CH}}_{4_{r}}$), mean ${\mathrm{CH}}_{4}$ concentration during eructation (${\mathrm{CH}}_{4_{e}}$), sum of ${\mathrm{CH}}_{4}$ concentrations per minute during respiration (${\mathrm{CH}}_{4_{\mathrm{rsum}}}$), sum of ${\mathrm{CH}}_{4}$ concentrations per minute during eructation (${\mathrm{CH}}_{4_{\mathrm{esum}}}$), maximal ${\mathrm{CH}}_{4}$ concentration during respiration (${\mathrm{CH}}_{4_{\mathrm{rmax}}}$), maximal ${\mathrm{CH}}_{4}$ concentration during eructation (${\mathrm{CH}}_{4_{\mathrm{emax}}}$), and eructation events per minute (${\mathrm{CH}}_{4_{\mathrm{event}}}$). Large levels of ewe ${\mathrm{CH}}_{4}$ emissions representing energy losses were significantly associated with lower LBW (P<0.05), lower EBW (P<0.01) and lower BFT (P<0.05). For genetic parameter estimations, we applied single- and multiple-trait animal models. Heritabilities and additive genetic variances for ${\mathrm{CH}}_{4}$ traits were small, i.e., heritabilities in the range from <0.01 (${\mathrm{CH}}_{4_{r+e}}$, ${\mathrm{CH}}_{4_{r}}$, ${\mathrm{CH}}_{4_{\mathrm{rmax}}}$, ${\mathrm{CH}}_{4_{\mathrm{esum}}}$) to 0.03 (${\mathrm{CH}}_{4_{\mathrm{rsum}}}$). We estimated negative genetic correlations between ${\mathrm{CH}}_{4}$ traits and EBW in the range from -0.44 (${\mathrm{CH}}_{4_{r+e}}$) to -0.05 (${\mathrm{CH}}_{4_{\mathrm{rsum}}}$). Most of the ${\mathrm{CH}}_{4}$ traits were genetically negatively correlated with BCS (-0.81 for ${\mathrm{CH}}_{4_{\mathrm{esum}}}$) and with BFT (-0.72 for ${\mathrm{CH}}_{4_{\mathrm{emax}}}$), indicating same genetic mechanisms for ${\mathrm{CH}}_{4}$ output and energy efficiency indicators. Addressing the intergenerational aspect, genetic correlations between ${\mathrm{CH}}_{4}$ emissions from ewes and LBW ranged between -0.35 (${\mathrm{CH}}_{4_{r+e}}$) and 0.01 (${\mathrm{CH}}_{4_{\mathrm{rsum}}}$, ${\mathrm{CH}}_{4_{\mathrm{rmax}}}$), indicating that breeding on reduced ${\mathrm{CH}}_{4}$ emissions (especially eructation traits) contribute to genetic improvements in lamb weaning performance.

## 1 Introduction

Methane (${\mathrm{CH}}_{4}$) is a by-product of microbial fermentation processes in ruminants (Henderson et al., 2015) and a potential greenhouse gas. Furthermore, ${\mathrm{CH}}_{4}$ emissions reflect an unused proportion of gross energy intake (Johnson and Ward, 1996; Baker, 1999). Fodder is the major cost factor in sheep production systems (Ellison et al., 2017). Hence, there is an increasing interest to breed animals with improved productivity and feed efficiency (i.e., feed intake in relation to body weight gain), possibly via selection on low individual ${\mathrm{CH}}_{4}$ emissions (Paganoni et al., 2017). Pickering et al. (2015) and Paganoni et al. (2017) indicated genetic variation and small to moderate heritabilities for ${\mathrm{CH}}_{4}$ traits in dairy cows and sheep, and Rösler et al. (2018) described an individual variation in enteric ${\mathrm{CH}}_{4}$ emissions in female goats. Furthermore, the economic benefits from selection scenarios including ${\mathrm{CH}}_{4}$ traits (Robinson and Oddy, 2016) suggest consideration of ${\mathrm{CH}}_{4}$ or of ${\mathrm{CH}}_{4}$ indicator traits into overall sheep breeding goals.

In this regard, respiration chamber calorimetry is the “golden standard” to determine ${\mathrm{CH}}_{4}$ emissions in sheep. Nevertheless, respiration chamber measurements imply strong efforts regarding logistics, associated with a substantial cost component. In consequence, only a small number of sheep can be phenotyped for ${\mathrm{CH}}_{4}$ using the respiration chamber technique. In addition, respiration chambers reflect an artificial environment, which is not representative of sheep kept in pasture-based production systems. Animals might show abnormal behavior (e.g., reduced dry matter intake, DMI) in the chamber, possibly influencing a ${\mathrm{CH}}_{4}$ emission pattern (Kabreab et al., 2006; Bickell et al., 2014). Thus, Knapp et al. (2014) and Huhtanen et al. (2015) requested alternative reliable and cost-efficient methods for ${\mathrm{CH}}_{4}$ recording, especially under field conditions. In such a context, approaches based on feed supplements were unsuitable under grazing conditions (Baker, 1999). Predictions of ${\mathrm{CH}}_{4}$ via deterministic modeling usually require a large amount of input data, e.g., DMI, dietary or milk components, which are difficult to record (Kabreab et al., 2006; Yin et al., 2015). Further indirect methods for ${\mathrm{CH}}_{4}$ emission predictions based on the ruminal microbiome composition but associations between ${\mathrm{CH}}_{4}$ production and microbiome characteristics were inconsistent (Shi et al., 2014; Ellison et al., 2017). The portable handheld laser methane detector (LMD) was suitable for ${\mathrm{CH}}_{4}$ recording in dairy cattle under field conditions, with low inter-observer variability (Chagunda et al., 2009b). In validations, correlations between LMD ${\mathrm{CH}}_{4}$ and ${\mathrm{CH}}_{4}$ measurements from the respiration calorimetric chamber were large (Chagunda and Yan, 2011).

With regard to associations between ${\mathrm{CH}}_{4}$ output and other breeding goal traits, Zetouni et al. (2018) estimated negative genetic correlations between ${\mathrm{CH}}_{4}$ production (g d${}^{-1}$) and body conformation traits in Danish Holstein cows. Nevertheless, there is a gap of knowledge addressing “across generation studies”, i.e., association analyses between indicators for energy balances of ewes (including ${\mathrm{CH}}_{4}$ emissions) and body weights of their lambs (LBW; also characterizing productivity of the ewe).

The objective of the present study was to focus on such intergenerational aspects, considering ${\mathrm{CH}}_{4}$ measurements from ewes (recorded via LMD) as energy balance indicators. The ${\mathrm{CH}}_{4}$ databases were used (i) to define and to evaluate different ${\mathrm{CH}}_{4}$ measurement characteristics, (ii) to estimate genetic parameters for ${\mathrm{CH}}_{4}$ measurements, and (iii) to correlate phenotypically and genetically ewe ${\mathrm{CH}}_{4}$ measurements with other breeding goal traits from a within- and transgenerational perspective.

## 2 Materials and methods

### 2.1 Ethics statement

The housing and treatment of the animals were carried out in accordance with national and international laws. The study was restricted to routine on-farm observations. All presented methods were non-invasive. Therefore, they did not cause the included animals pain, suffering or harm, in compliance with the German Animal Welfare Act §7. Nevertheless, the presented procedures have been approved for a subsample of ewes that were used for additional blood parameter analyses by the regional board of Giessen (V 54-19 c 20 15 h 01 GI 18/14 Nr. G 62/2017).

### 2.2 Production system

For trait recording, we focused on sheep from the University of Giessen research station “Oberer Hardthof”, reflecting a mixture of grazing (spring to fall) and high-input (fall to spring) sheep production system. The farm is located 200 m above sea level in the federal state Hesse in the middle of Germany. The average annual temperature is 8.8 ${}^{\circ }$C, and the average precipitation amount is 695 mm per year. The farm comprises 70 ha for a flock including 630 ewes, 7 rams and 98 hoggets of Merinoland (ML) and Rhön sheep (RH). During the lambing season, the flock was fed hay ad libitum. The hay quality was as follows: 90.3 % dry matter (DM), 40.2 % crude fiber (CF), 6.8 % crude protein (CP), 1.3 % crude lipid (EE) and 7.8 MJ metabolizable energy (ME) per kg in DM. Ewes within the last third of gestation received additional concentrates up to 1 kg d${}^{-1}$. The concentrates were composed of barley, wheat, rapeseed meal extract, wheat bran and triticale (6.8 % CF, 18 % CP, 2.6 % EE, 10.8 ME MJ per kg DM). The calculated daily ration for a twin-suckling ewe with an average body weight of 85 kg contained 1.8 kg hay and 900 g concentrates (21.84 MJ ME per ewe and day). Lambs had ad libitum access to concentrates at an age of 21 to 28 d. They were weaned group-wise at a mean age of 65.35±5.35 d with an average body weight of 26.10±4.91 kg.

### 2.3 Animals and traits

Data recording spanned a period from 2017 to 2018. The study considered 330 ewes (253 ML, 77 RH) and their purebred 629 lambs (478 ML, 151 RH). The age of ewes ranged from 22.1 to 96.8 months (mean = 51.3±18.2 months). In a subset of 177 ewes (133 ML, 44 RH), the whole pattern of traits was recorded: ewe body weight (EBW) (digital scale: model 703, TRU-TEST Group, Auckland, New Zealand), ewe body condition score (BCS), ewe backfat thickness (BFT) in millimeters (mm), and the individual ${\mathrm{CH}}_{4}$ concentrations (µL L${}^{-1}$) in the exhaled air. Body condition score was assessed by palpating the transverse and spinous processes of the lumbar region around the backbone. Scores ranged on a scale from 1.0 (emaciated) to 5.0 (obese) with increments of 0.5 (Russel et al., 1969). Backfat thickness was measured on the right side directly behind the 13th rib (Silva et al., 2006; Gernand and Lenz, 2005), using a mobile ultrasound transducer (EasiScan ultrasound scanner, 4.5–8.5 MHz linear, BCF Technology Ltd., Bellshill, Scotland). Individual ${\mathrm{CH}}_{4}$ concentrations in the exhaled air were measured using an LMD (Crowcon LaserMethane Mini, Tokyo Gas Engineering Co Ltd., Tokyo, Japan). Lamb body weight was recorded from 281 offspring (216 ML, 65 RH). A further subset for genetic analyses considered only EBW and BCS of an additional 153 ewes (120 ML, 33 RH) and LBW of their 348 lambs (262 ML, 86 RH). We generated a longitudinal data structure, implying ewe trait and LBW recording on the same days in intervals of 3 weeks from parturition until weaning.

### 2.4 Laser methane detector method and ${\mathrm{CH}}_{4}$ data preparation

According to Ricci et al. (2014), the interval between feeding and LMD ${\mathrm{CH}}_{4}$ recording comprised 3–5 h. Ricci et al. (2014) identified substantial impact of meteorological data on individual ${\mathrm{CH}}_{4}$ emissions. Consequently, we selected a wind-still environment, and we accounted for temperature and humidity in genetic-statistical modeling. In order to guarantee standardized trait recording, ewe ${\mathrm{CH}}_{4}$ measurements were performed after weighing in the weighing facility, and additionally an assistant fixated the ewes during ${\mathrm{CH}}_{4}$ recording. Hence, we always had a distance of exactly 1 m between the operator (i.e., the LMD) and the sheep's nostrils, and we avoided noisy data because of an uncontrolled movement (Ricci et al., 2014; Huhtanen et al., 2015).

**Figure 1:**
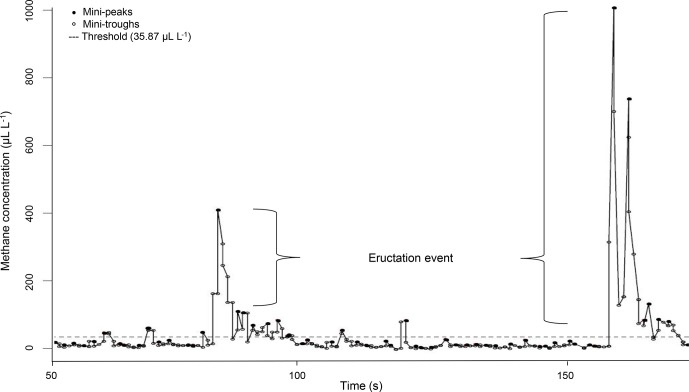
Example for a ${\mathrm{CH}}_{4}$ measurement profile of one ewe recorded via laser methane detector (LMD) and corrected for background ${\mathrm{CH}}_{4}$. Values under the threshold describe ${\mathrm{CH}}_{4}$ emissions during respiration and values above the threshold describe ${\mathrm{CH}}_{4}$ emissions during eructation.

The LMD recorded ${\mathrm{CH}}_{4}$ concentrations in intervals of 0.5 s in the exhaled air. Methane concentrations were directly displayed in parts per million-meter (ppm-m) (Tokyo Gas Engineering Co. Ltd., 2013). Because the distance between the LMD and the ewe's nostrils was exactly 1 m in the present study, the ${\mathrm{CH}}_{4}$ concentration was expressed in microliters per liter (Ricci et al., 2014). Ongoing ${\mathrm{CH}}_{4}$ data preparation in R 3.3.2 (R Development Core Team, 2016) is based on the protocol as suggested by Ricci et al. (2014): the minimum ${\mathrm{CH}}_{4}$ concentration of each measurement was set as a background ${\mathrm{CH}}_{4}$ concentration, i.e., to reflect environmental ${\mathrm{CH}}_{4}$ influence (overall mean background ${\mathrm{CH}}_{4}$ = 6.82 µL L${}^{-1}$). Afterwards, background ${\mathrm{CH}}_{4}$ was subtracted from the remaining ${\mathrm{CH}}_{4}$ records. Because the LMD detection is based on ${\mathrm{CH}}_{4}$ in the exhaled air, we considered the dynamics of the respiratory cycle (Chagunda, 2013). In this regard, Fig. 1 illustrates the ${\mathrm{CH}}_{4}$ measurement profile for one ewe. Every dot represents a detected ${\mathrm{CH}}_{4}$ concentration in microliters per liter. The ${\mathrm{CH}}_{4}$ emission profile represents small increases in ${\mathrm{CH}}_{4}$ concentration (mini-peaks; solid dots) due to exhalation or eructation. Before and after one mini-peak, mini-troughs (open dots) represent small ${\mathrm{CH}}_{4}$ concentration decreases due to ${\mathrm{CH}}_{4}$ diffusions. Only mini-peak data (solid dots) were log-transformed (natural logarithm) and used for further analyses (Chagunda et al., 2009b). Because mini-peaks reflect two different possibilities of ${\mathrm{CH}}_{4}$ excretion – (i) ${\mathrm{CH}}_{4}$ absorption from the rumen or lower digestive tract and emission via the lungs (respiration) and (ii) ${\mathrm{CH}}_{4}$ emissions directly from the rumen (eructation) (Murray et al., 1976) – a double normal distribution for mini-peaks was assumed. The dashed line in Fig. 1 shows the defined threshold at 95 % cumulative probability (35.87 µL L${}^{-1}$) for the lower normal distribution from all ${\mathrm{CH}}_{4}$ mini-peak observations. Consequently, all mini-peaks (solid dots) under the dashed line belong to ${\mathrm{CH}}_{4}$ emitted during respiration. All mini-peaks (solid dots) above the threshold represent ${\mathrm{CH}}_{4}$ concentrations during eructation. A group of solid dots including more than two mini-peaks above the dashed line was defined as one eructation event. Each normal distribution (respiration ${\mathrm{CH}}_{4}$; eructation ${\mathrm{CH}}_{4}$) represents a separate ${\mathrm{CH}}_{4}$ dataset with separate mean and maximum. Based on the data preparation protocol, the following ${\mathrm{CH}}_{4}$ traits were defined:

${\mathrm{CH}}_{4_{r+e}}$: mean ${\mathrm{CH}}_{4}$ concentration during respiration and eructation,
${\mathrm{CH}}_{4_{r}}$: mean ${\mathrm{CH}}_{4}$ concentration during respiration,
${\mathrm{CH}}_{4_{\mathrm{rsum}}}$: sum of ${\mathrm{CH}}_{4}$ concentrations per minute during respiration,
${\mathrm{CH}}_{4_{\mathrm{rmax}}}$: maximum ${\mathrm{CH}}_{4}$ concentration during respiration,
${\mathrm{CH}}_{4_{e}}$: mean ${\mathrm{CH}}_{4}$ concentration during eructation,
${\mathrm{CH}}_{4_{\mathrm{esum}}}$: sum of ${\mathrm{CH}}_{4}$ concentrations per minute during eructation,
${\mathrm{CH}}_{4_{\mathrm{emax}}}$: maximum ${\mathrm{CH}}_{4}$ concentration during eructation,
  ${\mathrm{CH}}_{4_{\mathrm{event}}}$: number of eructation events per minute.

Descriptive statistics for the defined ${\mathrm{CH}}_{4}$ traits are given in Table 1.

**Table 1:** Descriptive statistics for ewe body weight (EBW), ewe body condition score (BCS), ewe backfat thickness (BFT) and ${\mathrm{CH}}_{4}$ traits in the breeds Merinoland (ML) and Rhön sheep (RH).

Breed	Trait${}^{*}$	No.	Mean	SD	Median	Minimum	Maximum
ML	EBW (kg)	1133	94.5	11.5	94.5	57.0	130
	BCS	1133	3.35	0.78	3.50	1.00	5.00
	BFT (mm)	513	5.78	2.39	6.00	1.00	12.0
	${\mathrm{CH}}_{4_{r+e}}$ (µL L${}^{-1}$)	555	32.8	12.1	31.5	7.92	70.0
	${\mathrm{CH}}_{4_{r}}$ (µL L${}^{-1}$)	555	18.5	4.48	18.1	7.92	33.2
	${\mathrm{CH}}_{4_{\mathrm{rmax}}}$ (µL L${}^{-1}$)	555	33.8	1.82	35.0	25.0	35.0
	${\mathrm{CH}}_{4_{\mathrm{rsum}}}$ (µL L${}^{-1}$ min${}^{-1}$)	555	427	97.5	413	0.00	767
	${\mathrm{CH}}_{4_{e}}$ (µL L${}^{-1}$)	555	90.9	36.1	84.2	0.00	279
	${\mathrm{CH}}_{4_{\mathrm{emax}}}$ (µL L${}^{-1}$)	555	292	193	244	0.00	975
	${\mathrm{CH}}_{4_{\mathrm{esum}}}$ (µL L${}^{-1}$ min${}^{-1}$)	555	522	342	470	0.00	1865
	${\mathrm{CH}}_{4_{\mathrm{event}}}$ (no. min${}^{-1}$)	555	0.95	0.55	0.96	0.00	3.38
RH	EBW (kg)	360	70.6	8.36	69.5	54.0	92.5
	BCS	360	2.99	0.65	3.00	1.00	4.50
	BFT (mm)	183	6.38	1.56	6.00	2.00	11.0
	${\mathrm{CH}}_{4_{r+e}}$ (µL L${}^{-1}$)	175	31.4	12.0	30.1	7.03	67.5
	${\mathrm{CH}}_{4_{r}}$ (µL L${}^{-1}$)	175	16.5	3.92	16.7	7.03	27.6
	${\mathrm{CH}}_{4_{\mathrm{rmax}}}$ (µL L${}^{-1}$)	175	33.4	2.01	34.0	26.0	35.0
	${\mathrm{CH}}_{4_{\mathrm{rsum}}}$ (µL L${}^{-1}$ min${}^{-1}$)	175	381	84.2	375	196	796
	${\mathrm{CH}}_{4_{e}}$ (µL L${}^{-1}$)	175	93.8	36.7	87.2	0.00	242
	${\mathrm{CH}}_{4_{\mathrm{emax}}}$ (µL L${}^{-1}$)	175	277	181	230	0.00	893
	${\mathrm{CH}}_{4_{\mathrm{esum}}}$ (µL L${}^{-1}$ min${}^{-1}$)	175	513	335	463	0.00	1706
	${\mathrm{CH}}_{4_{\mathrm{event}}}$ (no. min${}^{-1}$)	175	0.99	0.58	0.99	0.00	3.67

${}^{*}$ ${\mathrm{CH}}_{4_{r+e}}$: mean ${\mathrm{CH}}_{4}$ concentration during respiration and eructation; ${\mathrm{CH}}_{4_{r}}$: mean ${\mathrm{CH}}_{4}$ concentration during respiration; ${\mathrm{CH}}_{4_{\mathrm{rmax}}}$: maximum ${\mathrm{CH}}_{4}$ concentration during respiration; ${\mathrm{CH}}_{4_{\mathrm{rsum}}}$: sum of ${\mathrm{CH}}_{4}$ concentrations per minute during respiration; ${\mathrm{CH}}_{4_{e}}$: mean ${\mathrm{CH}}_{4}$ concentration during eructation; ${\mathrm{CH}}_{4_{\mathrm{emax}}}$: maximum ${\mathrm{CH}}_{4}$ concentration during eructation; ${\mathrm{CH}}_{4_{\mathrm{esum}}}$: sum of ${\mathrm{CH}}_{4}$ concentrations per minute during eructation; ${\mathrm{CH}}_{4_{\mathrm{event}}}$: number of eructation events per minute.

### 2.5 Phenotypic associations between ewe ${\mathrm{CH}}_{4}$ emissions with ewe and lamb body weight traits

The impact of ewe ${\mathrm{CH}}_{4}$ emissions on EBW, BFT, BCS and LBW was studied via mixed model applications as implemented in the software package SAS Studio Version 3.71 (SAS Institute Inc., 2017). In matrix notation, the statistical model Eq. (1) was defined as follows:

1 y=Xb+Zu+e,

where y is a vector of observations for the traits EBW, BFT, BCS and LBW; b is a vector of fixed effects including the combined effect of birth type (single, twin, triplet) and sex of the lamb (male, female), breed (ML, RH), the combined month–of-the-year effect, ewe BCS (1–5) (apart from the models where BCS and BFT are the traits of interest), and the fixed regression of the lamb age (0 to 73 d) within breed modeled with Legendre polynomials of fourth order. Furthermore, vector b included in consecutive runs the different ${\mathrm{CH}}_{4}$ traits ${\mathrm{CH}}_{4_{r+e}}$- (≤25 µL L${}^{-1}$; 26–35 µL L${}^{-1}$; ≥36 µL L${}^{-1}$), ${\mathrm{CH}}_{4_{r}}$- (≤15.5 µL L${}^{-1}$; 15.6–19.5 µL L${}^{-1}$; > 19.5 µL L${}^{-1}$), ${\mathrm{CH}}_{4_{\mathrm{rsum}}}$- (≤360 µL L${}^{-1}$ min${}^{-1}$; 361–439 µL L${}^{-1}$ min${}^{-1}$; ≥440 µL L${}^{-1}$ min${}^{-1}$), ${\mathrm{CH}}_{4_{\mathrm{rmax}}}$- (≤34 µL L${}^{-1}$; > 34 µL L${}^{-1}$), ${\mathrm{CH}}_{4_{e}}$- (≤72 µL L${}^{-1}$; 73–99 µL L${}^{-1}$; > 99 µL L${}^{-1}$), ${\mathrm{CH}}_{4_{\mathrm{esum}}}$- (≤310 µL L${}^{-1}$ min${}^{-1}$; 311–620 µL L${}^{-1}$ min${}^{-1}$; > 620 µL L${}^{-1}$ min${}^{-1}$), ${\mathrm{CH}}_{4_{\mathrm{emax}}}$- (≤170 µL L${}^{-1}$; 171–315 µL L${}^{-1}$; > 315 µL L${}^{-1}$), and ${\mathrm{CH}}_{4_{\mathrm{event}}}$ class (≤0.96 min${}^{-1}$; > 0.96 min${}^{-1}$). u is a vector for the random ewe or lamb effect considering up to four repeated measurements per ewe and lamb, e is a vector of random residual effects, and X and Z are incidence matrices for b and u, respectively.

### 2.6 Genetic parameters for ewe ${\mathrm{CH}}_{4}$ emissions and body weight traits 

Genetic (co)variance components for all trait combinations including EBW, BCS, BFT and LBW were estimated by applying the software package DMU (Madsen and Jensen, 2013) and using the AI-REML algorithm for multiple-trait animal models. For the ${\mathrm{CH}}_{4}$ traits ${\mathrm{CH}}_{4_{r+e}}$, ${\mathrm{CH}}_{4_{r}}$, ${\mathrm{CH}}_{4_{\mathrm{rmax}}}$, ${\mathrm{CH}}_{4_{\mathrm{rsum}}}$, ${\mathrm{CH}}_{4_{e}}$, ${\mathrm{CH}}_{4_{\mathrm{emax}}}$, ${\mathrm{CH}}_{4_{\mathrm{esum}}}$ and ${\mathrm{CH}}_{4_{\mathrm{event}}}$, single-trait animal models were applied. Multiple-trait models converged properly for EBW, BCS, BFT and LBW due to the larger dataset, but some convergence problems occurred when additionally including ${\mathrm{CH}}_{4}$ traits from the smaller subset of phenotyped ewes. This was the reason for the application of single-trait animal models for ${\mathrm{CH}}_{4}$ traits.

The statistical model Eq. (2) for genetic analyses in matrix notation was defined as follows:

2 y=Xb+Za+Wpe+e,

where y is a vector of observations for EBW, BCS, BFT and LBW and ${\mathrm{CH}}_{4}$ traits; b is a vector of fixed effects including all effects as introduced in model (1) and the fixed effects for the temperature class (≤4, 4.1–8.5, 8.6–11, > 11 ${}^{\circ }$C) and for the humidity class (≤43 %, 44 %–55 %, 56 %–64 %, > 64 %); a is a vector of random additive genetic effects considering the genetic relationships from an animal model; pe is a vector of random permanent environmental effects for repeated measurements; e is a vector of random residual effects; and X, Z and W are incidence matrices for b, a and pe, respectively.

Correlations among estimated breeding values (EBVs) for ${\mathrm{CH}}_{4}$ traits (EBV from the single-trait models), and between EBV for ${\mathrm{CH}}_{4}$ traits and EBV for EBW, BCS, BFT and LBW (EBV from the multiple-trait model) were transformed into genetic correlations according to Calo et al. (1973):

$$r_{g1,\;2\;}=\frac{\sqrt{\left(\sum_{i}R_{i1}\right)\cdot \left(\sum_{i}R_{i2}\right)}}{\sum_{i}\left(R_{i1}\cdot R_{i2}\right)}\cdot r\left({\mathrm{EBV}}_{1},{\mathrm{EBV}}_{2}\right),$$

where R was the EBV reliability for an individual i in trait j. For the genetic correlation approximations, we only considered EBV from ewes with phenotypic records.

## 3 Results and discussion

### 3.1 Strategies of ${\mathrm{CH}}_{4}$ trait definitions 

The introduced ${\mathrm{CH}}_{4}$ data preparation strategy is very complex. Nevertheless, a separation of respiration and eructation ${\mathrm{CH}}_{4}$ is physiologically reasonable and considers environmental air movements. We identified a high agreement between statistically defined eructation events and ewe eructation during trait recording (own visual inspections of eructation events during ${\mathrm{CH}}_{4}$ recording). In our data, during the 3 min recording interval, 95 % of ewes eructated at least once. The eructation probability in the study by Ricci et al. (2014) was slightly lower (92 %), but they considered a 2 min recording interval. Hence, a minor disadvantage for specific ${\mathrm{CH}}_{4}$ eructation trait definitions is the small percentage of ewes (5 %) which had to be excluded from data processing. Chagunda et al. (2009b) introduced a further transformation of LMD output data (µL L${}^{-1}$) into daily ${\mathrm{CH}}_{4}$ production (g d${}^{-1}$) but without distinguishing into respiration and eructation. The data processing procedure by Chagunda et al. (2009b) also required complex equations including approximations for, for example, individual respiratory tidal volume or for the daily animal activity level. Methane traits as defined in our study reflect “pure” ${\mathrm{CH}}_{4}$ emissions, whereas ${\mathrm{CH}}_{4}$ predictions by Chagunda et al. (2009b) depend on body trait or physiological characteristics. Hence, in quantitative genetic studies, and following a deterministic ${\mathrm{CH}}_{4}$ prediction strategy, the estimated heritability does not fully reflect the individual ${\mathrm{CH}}_{4}$ genetic background (Yin et al., 2015). In genome-wide association studies, Manzanilla-Pech et al. (2016) found an overlap for 19 % of SNP markers being significantly associated with DMI, body weight and individual ${\mathrm{CH}}_{4}$ production in dairy cattle. In consequence, they suggested consideration of residual ${\mathrm{CH}}_{4}$ emissions that are additionally pre-corrected for ${\mathrm{CH}}_{4}$ indicator traits (e.g., for DMI and for body weight).

Moreover, our ${\mathrm{CH}}_{4}$ trait separation into respiration and eructation provides deeper insights into the different physiological mechanisms associated with ${\mathrm{CH}}_{4}$ output. The ${\mathrm{CH}}_{4}$ separation strategy allows studying the isolated influence of either respiration or eructation on ewe body condition traits and on LBW. Nevertheless, our approach depends on the individual threshold definition for the two normal distributions (respiration and eructation).

**Figure 2:**
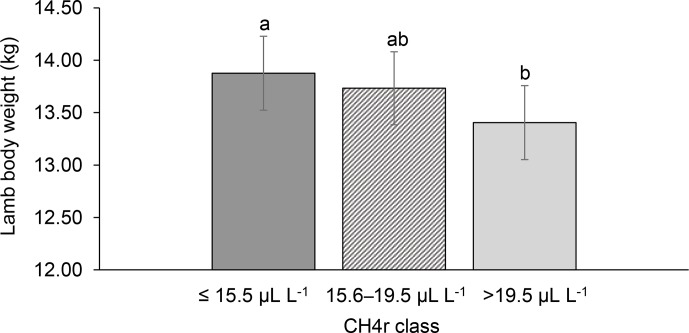
Least-squares means for lamb body weight (LBW) depending on ewe mean ${\mathrm{CH}}_{4}$ concentration during respiration (${\mathrm{CH}}_{4_{r}}$ class; model 1). Different letters represent significant differences (P<0.01).

### 3.2 Phenotypic impact of ${\mathrm{CH}}_{4}$ traits on lamb body weight and ewe body condition

**Figure 3:**
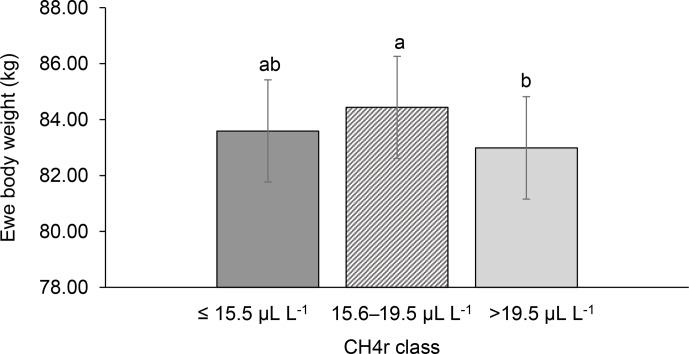
Least-squares means for ewe body weight (EBW) depending on ewe mean ${\mathrm{CH}}_{4}$ concentration during respiration (${\mathrm{CH}}_{4_{r}}$ class; model 1). Different letters represent significant differences (P<0.01).

Among all ${\mathrm{CH}}_{4}$ traits, the inclusion of ${\mathrm{CH}}_{4}$ mean concentration during respiration (${\mathrm{CH}}_{4_{r}}$) as class effect in model (1) gave the lowest value for the Akaike information criterion (Akaike, 1973) (Table 2). Hence, ${\mathrm{CH}}_{4_{r}}$ consideration indicated statistical modeling superiority. The ${\mathrm{CH}}_{4_{r}}$ class effect significantly influenced LBW (P<0.05) and EBW (P<0.01) (model 1). Ewes with low mean ${\mathrm{CH}}_{4}$ emissions during respiration reared heavier lambs than ewes with high ${\mathrm{CH}}_{4}$ emissions (P<0.001; Fig. 2). Simultaneously, low mean ${\mathrm{CH}}_{4}$ emissions during respiration were associated with larger estimates for EBW during lactation (P<0.001; Fig. 3). EBW during lactation for ewes from the low ${\mathrm{CH}}_{4_{\mathrm{rmax}}}$ class was significantly higher (0.74 kg, P<0.05) compared to EBW from ewes with high ${\mathrm{CH}}_{4_{\mathrm{rmax}}}$ emissions (${\mathrm{CH}}_{4_{\mathrm{rmax}}}$ class > 34 µL L${}^{-1}$). In cattle, Johnson and Johnson (1995) identified high ${\mathrm{CH}}_{4}$ emissions as major contributors to energy losses, comprising 5 %–12 % of the gross energy intake. Consequently, limited energy is available for milk production, explaining the lower LBW of lambs from ewes with high ${\mathrm{CH}}_{4}$ output during lactation. Kandel et al. (2017) and Chagunda et al. (2009a) confirmed such unfavorable associations between ${\mathrm{CH}}_{4}$ emissions and milk yield in cattle. Interestingly, the ${\mathrm{CH}}_{4}$ eructation traits represent larger ${\mathrm{CH}}_{4}$ emissions than the respiration traits (Blaxter and Joyce, 1963), but only the respiration ${\mathrm{CH}}_{4}$ traits ${\mathrm{CH}}_{4_{r}}$ and ${\mathrm{CH}}_{4_{\mathrm{rmax}}}$ significantly influenced LBW and EBW. An explanation for the significant impact of “low-level ${\mathrm{CH}}_{4}$” (${\mathrm{CH}}_{4_{r}}$, ${\mathrm{CH}}_{4_{\mathrm{rmax}}}$) on LBW and EBW might be due to the short recording interval of only 3 min. For a small recording interval, the percentage of respiration in relation to eructation is larger, compared to, for example, accumulate 24 h measurements.

**Table 2:** Akaike information criterion (AIC) for model (1) with the dependent traits of lamb body weight (LBW) or ewe body weight (EBW), considering different ${\mathrm{CH}}_{4}$ class effects.

	AIC	
${\mathrm{CH}}_{4}$ class	LBW	EBW
effect${}^{*}$		
${\mathrm{CH}}_{4_{r+e}}$	4567	4484
${\mathrm{CH}}_{4_{r}}$	4564	4476
${\mathrm{CH}}_{4_{\mathrm{rmax}}}$	4571	4485
${\mathrm{CH}}_{4_{\mathrm{rsum}}}$	4569	4487
${\mathrm{CH}}_{4_{e}}$	4571	4488
${\mathrm{CH}}_{4_{\mathrm{esum}}}$	4572	4488
${\mathrm{CH}}_{4_{\mathrm{emax}}}$	4572	4488
${\mathrm{CH}}_{4_{\mathrm{event}}}$	4571	4488

${}^{*}$ ${\mathrm{CH}}_{4_{r+e}}$: mean ${\mathrm{CH}}_{4}$ concentration during respiration and eructation; ${\mathrm{CH}}_{4_{r}}$: mean ${\mathrm{CH}}_{4}$ concentration during respiration; ${\mathrm{CH}}_{4_{\mathrm{rmax}}}$: maximum ${\mathrm{CH}}_{4}$ concentration during respiration; ${\mathrm{CH}}_{4_{\mathrm{rsum}}}$: sum of ${\mathrm{CH}}_{4}$ concentrations per minute during respiration; ${\mathrm{CH}}_{4_{e}}$: mean ${\mathrm{CH}}_{4}$ concentration during eructation; ${\mathrm{CH}}_{4_{\mathrm{emax}}}$: maximum ${\mathrm{CH}}_{4}$ concentration during eructation; ${\mathrm{CH}}_{4_{\mathrm{esum}}}$: sum of ${\mathrm{CH}}_{4}$ concentrations per minute during eructation; ${\mathrm{CH}}_{4_{\mathrm{event}}}$: number of eructation events per minute.

**Figure 4:**
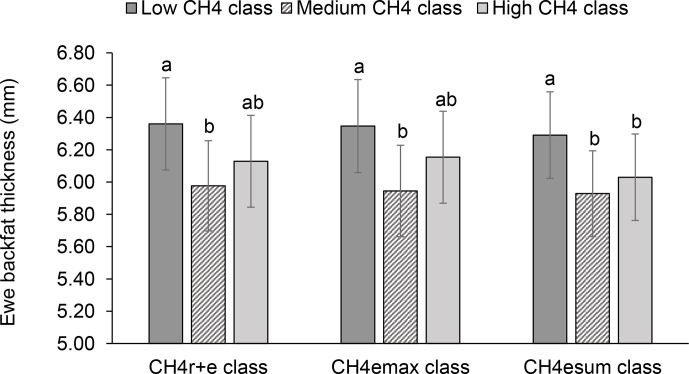
Least-squares means for ewe backfat thickness (BFT) depending on ewe mean ${\mathrm{CH}}_{4}$ concentration during respiration and eructation (${\mathrm{CH}}_{4_{r+e}}$ class), maximal ${\mathrm{CH}}_{4}$ concentration during eructation (${\mathrm{CH}}_{4_{\mathrm{emax}}}$ class), and sum of ${\mathrm{CH}}_{4}$ concentrations per minute during eructation (${\mathrm{CH}}_{4_{\mathrm{esum}}}$ class) (model 1). Definition of ${\mathrm{CH}}_{4}$ classes: low ${\mathrm{CH}}_{4}$ class: ${\mathrm{CH}}_{4_{r+e}}$ ≤25 µL L${}^{-1}$; ${\mathrm{CH}}_{4_{\mathrm{emax}}}$ ≤170 µL L${}^{-1}$; ${\mathrm{CH}}_{4_{\mathrm{esum}}}$ ≤310 µL L${}^{-1}$ min${}^{-1}$; medium ${\mathrm{CH}}_{4}$ class: ${\mathrm{CH}}_{4_{r+e}}$ 26–35 µL L${}^{-1}$; ${\mathrm{CH}}_{4_{\mathrm{emax}}}$ 171–315 µ L L${}^{-1}$; ${\mathrm{CH}}_{4_{\mathrm{esum}}}$ 311–620 µL L${}^{-1}$ min${}^{-1}$; high ${\mathrm{CH}}_{4}$ class: ${\mathrm{CH}}_{4_{r+e}}$ ≥36 µL L${}^{-1}$; ${\mathrm{CH}}_{4_{\mathrm{emax}}}$ > 315 µL L${}^{-1}$; ${\mathrm{CH}}_{4_{\mathrm{esum}}}$ > 620 µL L${}^{-1}$ min${}^{-1}$. Different letters represent significant differences (P<0.01).

Least-squares means for BFT declined with increasing ewe ${\mathrm{CH}}_{4}$ emissions. In this regard, ewes representing the medium ${\mathrm{CH}}_{4_{r+e}}$, ${\mathrm{CH}}_{4_{\mathrm{emax}}}$ and ${\mathrm{CH}}_{4_{\mathrm{esum}}}$ class had 0.38 to 0.43 mm less BFT than ewes from the low ${\mathrm{CH}}_{4}$ classes (P<0.05) (Fig. 4). An increase of ${\mathrm{CH}}_{4}$ emissions was associated with inefficient feed conversion, both contributing to energy deficiency during the early lactation stage (Hegarty et al., 2007; Paganoni et al., 2017). Hence, for energy deficiency compensation due to mammary requirements during lactation (intensified through ${\mathrm{CH}}_{4}$ emissions), ewes are forced to increase the mobilization rate of their own body fat depots (Bell, 1995), explaining the EBW and BFT decline. Such initiated catabolic processes depend on liver glycogen levels, which represent an important glucose (energy) body resource. Physiologically, catecholamine and glucagon blood levels are increasing, initiating the hydrolysis of body fat deposits (triglycerides) (Lawrence and Fowler, 2002). Ewes from the present study received concentrates but also responded with a BFT decline during lactation. Weston (1996) indicated the general problem of energy deficiency of lactating ewes, especially in pasture based production systems. Consequently, we suggest selection strategies on low ${\mathrm{CH}}_{4}$ emissions, in order to avoid further energy losses.

Bielak et al. (2016) suggested plasma levels of non-esterified fatty acids (NEFAs) as indicators for body fat mobilization. In lactating dairy cows, Bielak et al. (2016) identified a negative relationship between ${\mathrm{CH}}_{4}$ production per DMI and NEFA plasma levels. Nevertheless, intensified body fat mobilization with decreasing ${\mathrm{CH}}_{4}$ emissions in cows is in contradiction with the identified associations in the present study for sheep. Summarizing the phenotypic relationships, low values for ${\mathrm{CH}}_{4_{r+e}}$, ${\mathrm{CH}}_{4_{r}}$, ${\mathrm{CH}}_{4_{\mathrm{rmax}}}$, ${\mathrm{CH}}_{4_{\mathrm{emax}}}$ and ${\mathrm{CH}}_{4_{\mathrm{esum}}}$ in ewes were favorably associated with maternal body fat storage during lactation, and with increasing LBW.

### 3.3 Genetic parameters for ${\mathrm{CH}}_{4}$, ewe body condition traits and lamb body weight

In previous studies, variation of individual ${\mathrm{CH}}_{4}$ emissions was due to the diet composition and feeding system characteristics (Chagunda et al., 2009a; Pinares-Patiño et al., 2011; Bell et al., 2016), ruminal microbiome (Shi et al., 2014) and host genetic compositions (Pinares-Patiño et al., 2013). Genetic variation for ${\mathrm{CH}}_{4}$ emissions indicates the general possibilities for genetic selection, but this variation was only detected for ${\mathrm{CH}}_{4_{\mathrm{rsum}}}$ and ${\mathrm{CH}}_{4_{\mathrm{emax}}}$ (Table 3). Correspondingly, heritabilities for ${\mathrm{CH}}_{4}$ traits (Table 3) were close to zero, with the largest estimate for ${\mathrm{CH}}_{4_{\mathrm{rsum}}}$ (0.03). Pickering et al. (2015) and Paganoni et al. (2017) estimated heritabilities for ${\mathrm{CH}}_{4}$ in a comparable range from 0.05 to 0.14 in dairy cattle and sheep, respectively. For ${\mathrm{CH}}_{4}$ recordings, Paganoni et al. (2017) used portable accumulation chambers, and they applied the technique to lambs at post-weaning age and hoggets. Hence, ${\mathrm{CH}}_{4}$ heritabilities in ruminants are generally quite low, irrespective of the utilized measurement technology and the age of animals. Quite large residual variances (as also indicated in Table 3 for the traits in the present study) due to further environmental effects, which were not considered in statistical modeling, e.g., the individual stress level during measurement or the exhalation rate (Wu et al., 2018), might explain the generally small ${\mathrm{CH}}_{4}$ heritabilities in sheep. Large residual variances and small heritabilities indicate only minor selection response when aiming on reduced ${\mathrm{CH}}_{4}$ emissions. Besides, some ewes did not show any eructation during short time measurements. For the inclusion of eructation ${\mathrm{CH}}_{4}$ traits (${\mathrm{CH}}_{4_{e}}$, ${\mathrm{CH}}_{4_{\mathrm{emax}}}$, ${\mathrm{CH}}_{4_{\mathrm{esum}}}$, ${\mathrm{CH}}_{4_{\mathrm{event}}}$) into overall breeding goals, it is imperative to consider repeated measurements per animal, in order to guarantee at least one eructation per measurement.

**Table 3:** Heritabilities (h${}^{2}$) with standard errors (SE), additive genetic variances ($\sigma_{a}^{2}$), permanent environmental variances ($\sigma_{\mathrm{pe}}^{2}$) and residual variances ($\sigma_{e}^{2}$) for lamb body weight (LBW), ewe body weight (EBW), ewe body condition score (BCS), ewe backfat thickness (BFT) and ${\mathrm{CH}}_{4}$ traits.

		Variance component		
Trait${}^{*}$	h${}^{2}$ (SE)	$\sigma_{a}^{2}$	$\sigma_{\mathrm{pe}}^{2}$	$\sigma_{e}^{2}$
LBW (kg)	0.37 (0.16)	3.29	1.64	3.12
EBW (kg)	0.56 (0.12)	51.9	27.8	13.6
BCS	0.37 (0.10)	0.16	0.11	0.16
BFT (mm)	0.25 (0.13)	0.74	0.62	1.62
${\mathrm{CH}}_{4_{r+e}}$ (µL L${}^{-1}$)	0.00 (0.04)	< 0.01	6.12	132
${\mathrm{CH}}_{4_{r}}$ (µL L${}^{-1}$)	0.00 (0.04)	< 0.01	0.23	14.7
${\mathrm{CH}}_{4_{\mathrm{rsum}}}$ (µL L${}^{-1}$ min${}^{-1}$)	0.03 (0.04)	185	0.00	7125
${\mathrm{CH}}_{4_{\mathrm{rmax}}}$ (µL L${}^{-1}$)	0.00 (0.04)	< 0.01	0.00	3.08
${\mathrm{CH}}_{4_{e}}$ (µL L${}^{-1}$)	0.01 (0.04)	8.46	38.9	1049
${\mathrm{CH}}_{4_{\mathrm{esum}}}$ (µL L${}^{-1}$ min${}^{-1}$)	0.00 (0.04)	54.4	4051	105 677
${\mathrm{CH}}_{4_{\mathrm{emax}}}$ (µL L${}^{-1}$)	0.01 (0.04)	408	957	3236
${\mathrm{CH}}_{4_{\mathrm{event}}}$ (no. min${}^{-1}$)	0.02 (0.05)	0.01	0.02	0.25

${}^{*}$ ${\mathrm{CH}}_{4_{r+e}}$: mean ${\mathrm{CH}}_{4}$ concentration during respiration and eructation; ${\mathrm{CH}}_{4_{r}}$: mean ${\mathrm{CH}}_{4}$ concentration during respiration; ${\mathrm{CH}}_{4_{\mathrm{rmax}}}$: maximum ${\mathrm{CH}}_{4}$ concentration during respiration; ${\mathrm{CH}}_{4_{\mathrm{rsum}}}$: sum of ${\mathrm{CH}}_{4}$ concentrations per minute during respiration; ${\mathrm{CH}}_{4_{e}}$: mean ${\mathrm{CH}}_{4}$ concentration during eructation; ${\mathrm{CH}}_{4_{\mathrm{emax}}}$: maximum ${\mathrm{CH}}_{4}$ concentration during eructation; ${\mathrm{CH}}_{4_{\mathrm{esum}}}$: sum of ${\mathrm{CH}}_{4}$ concentrations per minute during eructation; ${\mathrm{CH}}_{4_{\mathrm{event}}}$: number of eructation events per minute.

Heritabilities for body condition traits were 0.56 for EBW, 0.37 for BCS, 0.25 for BFT and 0.37 for LBW (Table 3). Pinares-Patiño et al. (2013) and Borg et al. (2009) estimated similar heritabilities for live weight of 0.46 and 0.38, respectively. Jonker et al. (2018) estimated a heritability of 0.35 for LBW at 4 to 13 months of age, confirming our estimate of 0.37. The BFT heritability reflects estimates by Gernand et al. (2008) and Brito et al. (2017), but in both studies, the authors considered records from lambs instead of ewe traits.

**Table 4:** Genetic correlations between lamb body weight (LBW), ewe body weight (EBW), ewe body condition score (BCS) and ewe backfat thickness (BFT) with standard errors (in brackets), and approximated genetic correlations between LBW, EBW, BCS, BFT and ${\mathrm{CH}}_{4}$ traits.

	Genetic correlations			
Trait${}^{*}$	LBW (kg)	EBW (kg)	BCS	BFT (mm)
LBW (kg)		0.78 (0.14)	0.52 (0.23)	0.67 (0.29)
EBW (kg)			0.78 (0.09)	0.79 (0.18)
BCS				0.96 (0.13)
BFT (mm)				
${\mathrm{CH}}_{4_{r+e}}$ (µL L${}^{-1}$)	-0.35	-0.44	-0.42	-0.67
${\mathrm{CH}}_{4_{r}}$ (µL L${}^{-1}$)	-0.07	-0.14	0.10	0.05
${\mathrm{CH}}_{4_{\mathrm{rsum}}}$ (µL L${}^{-1}$ min${}^{-1}$)	0.01	-0.05	0.28	0.12
${\mathrm{CH}}_{4_{\mathrm{rmax}}}$ (µL L${}^{-1}$)	0.01	-0.35	-0.20	-0.08
${\mathrm{CH}}_{4_{e}}$ (µL L${}^{-1}$)	-0.17	-0.27	-0.34	-0.51
${\mathrm{CH}}_{4_{\mathrm{esum}}}$ (µL L${}^{-1}$ min${}^{-1}$)	-0.28	-0.32	-0.81	-0.49
${\mathrm{CH}}_{4_{\mathrm{emax}}}$ (µL L${}^{-1}$)	-0.18	-0.34	-0.30	-0.72
${\mathrm{CH}}_{4_{\mathrm{event}}}$ (no. min${}^{-1}$)	-0.22	-0.23	-0.44	-0.32

${}^{*}$ ${\mathrm{CH}}_{4_{r+e}}$: mean ${\mathrm{CH}}_{4}$ concentration during respiration and eructation; ${\mathrm{CH}}_{4_{r}}$: mean ${\mathrm{CH}}_{4}$ concentration during respiration; ${\mathrm{CH}}_{4_{\mathrm{rmax}}}$: maximum ${\mathrm{CH}}_{4}$ concentration during respiration; ${\mathrm{CH}}_{4_{\mathrm{rsum}}}$: sum of ${\mathrm{CH}}_{4}$ concentrations per minute during respiration; ${\mathrm{CH}}_{4_{e}}$: mean ${\mathrm{CH}}_{4}$ concentration during eructation; ${\mathrm{CH}}_{4_{\mathrm{emax}}}$: maximum ${\mathrm{CH}}_{4}$ concentration during eructation; ${\mathrm{CH}}_{4_{\mathrm{esum}}}$: sum of ${\mathrm{CH}}_{4}$ concentrations per minute during eructation; ${\mathrm{CH}}_{4_{\mathrm{event}}}$: number of eructation events per minute.

### 3.4 Genetic relationships between ${\mathrm{CH}}_{4}$ traits with ewe body condition traits and with lamb body weight

For the definition of overall sheep breeding goals including ${\mathrm{CH}}_{4}$, knowledge about genetic correlations and covariances with other economically important traits (e.g., LBW) is imperative. Genetic correlations between ${\mathrm{CH}}_{4}$ traits and EBW were slightly (${\mathrm{CH}}_{4_{r}}$, ${\mathrm{CH}}_{4_{\mathrm{rsum}}}$) or moderately negative (${\mathrm{CH}}_{4_{r+e}}$, ${\mathrm{CH}}_{4_{\mathrm{rmax}}}$, ${\mathrm{CH}}_{4_{e}}$, ${\mathrm{CH}}_{4_{\mathrm{emax}}}$, ${\mathrm{CH}}_{4_{\mathrm{esum}}}$, ${\mathrm{CH}}_{4_{\mathrm{event}}}$), indicating that breeding on low ${\mathrm{CH}}_{4}$ emissions increases EBW and vice versa (Table 4). Generally, genetic correlation estimates between ${\mathrm{CH}}_{4}$ traits and EBW were in agreement with the phenotypic associations from model (1). In contrast to our results, Pinares-Patiño et al. (2013) estimated a differing genetic correlation of 0.80 between ${\mathrm{CH}}_{4}$ production (g d${}^{-1}$) and body weights of lambs at the age of 8 months. In the present study, BCS was moderately negatively correlated with all ${\mathrm{CH}}_{4}$ traits reflecting eructation (${\mathrm{CH}}_{4_{e}}$, ${\mathrm{CH}}_{4_{\mathrm{emax}}}$, ${\mathrm{CH}}_{4_{\mathrm{esum}}}$, ${\mathrm{CH}}_{4_{\mathrm{event}}}$; Table 4). Also, genetic correlations between BCS with ${\mathrm{CH}}_{4_{r+e}}$ and ${\mathrm{CH}}_{4_{\mathrm{rmax}}}$ were negative. Zetouni et al. (2018) estimated a similar genetic correlation of -0.28 between ${\mathrm{CH}}_{4}$ production and BCS in Danish Holstein cows. Regarding BFT, a decline in ${\mathrm{CH}}_{4_{r+e}}$, ${\mathrm{CH}}_{4_{e}}$, ${\mathrm{CH}}_{4_{\mathrm{emax}}}$, ${\mathrm{CH}}_{4_{\mathrm{esum}}}$ and ${\mathrm{CH}}_{4_{\mathrm{event}}}$ was genetically associated with an incline in BFT. The genetic correlations between the respiration ${\mathrm{CH}}_{4}$ traits (${\mathrm{CH}}_{4_{r}}$, ${\mathrm{CH}}_{4_{\mathrm{rmax}}}$, ${\mathrm{CH}}_{4_{\mathrm{rsum}}}$) and BFT were close to zero. In contrast to the phenotypic associations, genetic relationships between respiration ${\mathrm{CH}}_{4}$ traits (${\mathrm{CH}}_{4_{r}}$, ${\mathrm{CH}}_{4_{\mathrm{rsum}}}$, ${\mathrm{CH}}_{4_{\mathrm{rmax}}}$) and LBW were quite low in the range from -0.07 to 0.01. On a genetic basis, among all ${\mathrm{CH}}_{4}$ traits, ${\mathrm{CH}}_{4_{r+e}}$ had the strongest genetic correlation (-0.35) with LBW.

Genetic correlations among EBW, BCS, BFT and LBW were positive, indicating an incline in LBW when selecting heavy ewes with high values for BCS and BFT. However, a strict selection on increasing EBW, BCS and BFT for indirect improvements of LBW might be associated with insulin resistance and hormone dysregulation in the future F1 and F2 generations (Pankey et al., 2017). Furthermore, adipose ewes were susceptible for dystocia (Peel et al., 2012).

In summary, the ${\mathrm{CH}}_{4}$ traits ${\mathrm{CH}}_{4_{r+e}}$, ${\mathrm{CH}}_{4_{e}}$, ${\mathrm{CH}}_{4_{\mathrm{esum}}}$, ${\mathrm{CH}}_{4_{\mathrm{emax}}}$ and ${\mathrm{CH}}_{4_{\mathrm{event}}}$ were genetically favorably correlated with LBW, indicating an increase in LBW and simultaneously improvements of EBW, BCS and BFT when selecting on low ${\mathrm{CH}}_{4}$ emissions, particularly during eructation. Nevertheless, small ${\mathrm{CH}}_{4}$ heritabilities indicate only slight selection response. Hence, in breeding goals or selection indices, it is imperative to consider the low heritability ${\mathrm{CH}}_{4}$ traits with high economic values (König et al., 2009).

## 4 Conclusions

${\mathrm{CH}}_{4}$ recording via LMD technique was successfully implemented in sheep under field conditions. On a longitudinal trait basis, we developed statistical strategies for distinguishing ${\mathrm{CH}}_{4}$ emissions in respiration and eructation. Large ewe ${\mathrm{CH}}_{4}$ emissions during respiration were associated with lower EBW as well as with impaired body weight development of their lambs. Additionally, a significant ewe BCS and BFT decrease after lambing was detected in ewes with high levels of ${\mathrm{CH}}_{4}$ emissions during eructation. Heritabilities for ${\mathrm{CH}}_{4}$ traits were close to zero ($h^{2}$ <0.01 to 0.03). Nevertheless, the genetic correlations between ${\mathrm{CH}}_{4}$ traits ${\mathrm{CH}}_{4_{r+e}}$, ${\mathrm{CH}}_{4_{e}}$, ${\mathrm{CH}}_{4_{\mathrm{emax}}}$, ${\mathrm{CH}}_{4_{\mathrm{esum}}}$ and ${\mathrm{CH}}_{4_{\mathrm{event}}}$ and energy efficiency indicators (e.g., LBW) suggest consideration of ewe ${\mathrm{CH}}_{4}$ emissions in overall sheep breeding goals when aspiring to feed efficiency improvements. We proved that the utilization of LMD equipment is an appropriate non-invasive method to measure ${\mathrm{CH}}_{4}$ emissions in sheep rapidly, easily and cost-efficiently. Furthermore, the differentiation between respiration and eructation ${\mathrm{CH}}_{4}$ emissions provides insights into physiological dynamics of ${\mathrm{CH}}_{4}$ emissions. Nevertheless, environmental (e.g., micrometeorology) and physiological (e.g., respiratory volume, behavior) factors can influence results from the applied ${\mathrm{CH}}_{4}$ recording technique and should be considered in future statistical modeling approaches.

## Data Availability

The data that support the findings of this study are available from the authors upon reasonable request.
